# Supplementary material for: Metric learning on expression data for gene function prediction
Source: Bioinformatics. 2019 Sep 28;36(4):1182–90. doi: 10.1093/bioinformatics/btz731 (PMC7703756; doi:10.1093/bioinformatics/btz731)
Supplement: btz731_Supplementary_Data [file btz731_supplementary_data.pdf]

# Metric Learning on Expression Data for Gene Function Prediction

Supplementary Material

Stavros Makrodimitris [1,2,*], Marcel J.T. Reinders [2,3] and Roeland C.H.J. van Ham [1,2]

[1]Delft Bioinformatics Lab, Delft University of Technology, Delft, the Netherlands,
[2]Keygene N.V., Wageningen, the Netherlands and
[3]Leiden Computational Biology Center, Leiden University Medical Center, Leiden, the Netherlands.
*To whom correspondence should be addressed.

## Contents

**1 *MLC* can filter out low-quality samples** **2**

**2 Use of ComBat with batches containing only 1 sample** **4**

**3 Competing Methods** **5**
- 3.1 Mutual Rank (*MR*) . . . . . 5
- 3.2 *GAAWGEFA* . . . . . 5

**4 Evaluation Modes** **6**
- 4.1 Cross-Validation Experiment . . . . . 6
- 4.2 CAFA3 Experiment . . . . . 6
- 4.3 CAFA-$\pi$ . . . . . 7

**5 Evaluation Measures** **8**
- 5.1 Unweighted and Weighted term-centric ROC AUC . . . . . 8
- 5.2 Protein-Centric Measures . . . . . 8

**6 Protein-Centric Results** **9**

**7 Statistical Significance of Differences in Cross-Validation Performance** **10**

**8 *MLC* does not pick up artificial information** **11**

**9 False positive rates for general and specific terms** **12**

**10 Performance as a function of term specificity** **13**

**11 Only reducing the number of samples does not boost *PCC* performance** **18**

**12 Comparison of *GAAWGEFA* and *MLC* weights** **19**

**13 Samples from the same study tend to get either selected or not selected together** **21**

**14 Evaluation on simulated data** **22**

**15 Weight profile** **24**

**16 The weights learned by *MLC* are consistent with the ontology structure and the existing annotations** **25**

**17 CAFA-$\pi$ Results** **27**

# 1 *MLC* can filter out low-quality samples

As mentioned in section 2.1 of the main document, as a pre-processing step we removed samples with fewer than 10 million reads mapped and applied ComBat to reduce the technical variation among samples. As a side experiment, we ran *MLC* using all the samples, i.e. bypassing the coverage filter, to test whether *MLC* will preferentially set the weights of these supposedly lower-quality samples to 0.

We ran *MLC* again for all GO terms including the whole double-loop cross-validation scheme, but now using all 4,215 samples. We removed 7 samples that were in "singleton" batches in order to be able to run ComBat (section 2.1, main document and Supplementary Material 2), leaving us with 4,208 samples. Then, we ranked the samples based on the weight values they get assigned for each term. The sample with lowest weight gets a rank of 0 and the one with the highest 4,207. If multiple samples (for instance say 1,000) get a weight value of 0, then all these samples get a rank of 0 and the immediately next sample gets a rank of 1,000. Other ties are handled similarly. Then, we used the median of each sample's ranks over all GO terms as a measure of how often it is selected by *MLC* (the higher the rank, the more often a sample is selected).

We found that *MLC* preferentially selected samples from the ones we had initially removed (left panel of Figure S1). We found that samples with about 1 million to 100 million reads have a similar distribution of median ranks, but samples with lower coverage tend to have larger median ranks. More specifically, for 188 out of 226 the terms for which *MLC* selected fewer than 500 samples, we found significant enrichment of the low-coverage samples using Fisher's exact test. Despite this, the performance of MLC was not affected (mean of 0.72), neither that of *MR* (also mean of 0.72). On the contrary, the performance of *PCC* slightly increased, from 0.69 to 0.7, remaining significantly worse than the other two methods.

However, when we removed the ComBat from our pipeline and repeated the whole experiment again, we found that the vast majority of samples with fewer than 1 million reads had a median rank of 0 (right panel of Figure S1). In that case, *MLC* suffered a performance drop, performing equal to *PCC* (mean *ROCAUC* of 0.7). Despite this, *MLC* remained superior on specific terms (Spearman $\rho$ between % improvement and term information content = 0.26).

This shows that – indeed – our method can identify poor-quality samples and avoid selecting them.

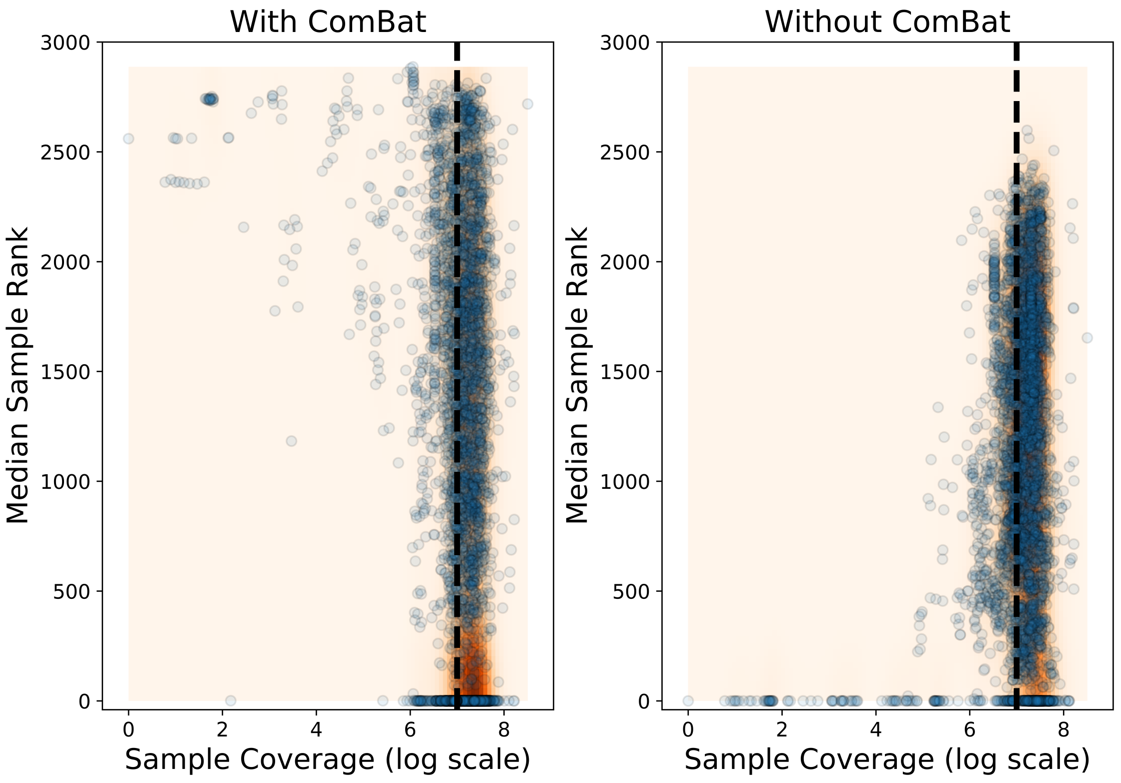


Figure S1: Sample median weight rank ($y$ axis) as a function of the total number of reads ($x$ axis, $log_{10}$ scale) when using ComBat (left) and when not using ComBat (right). The threshold we initially used to filter out samples is shown as black dashed line. Samples are shown as blue dots. For each GO term, we sort the $MLC$ weights and convert them to rank values. Then, for each sample, we plot the median of its ranks over all GO terms. The 2-dimensional density of the points as estimated using Gaussian kernels is also shown, with darker areas corresponding to higher density.

## 2 Use of ComBat with batches containing only 1 sample

If a batch contains only one sample, the variance of a gene within the samples of the batch is not defined, in which case ComBat only standardizes the means. Since different batches have widely different read counts, samples from batches with higher average coverage will contribute considerably more to the total variance of each gene's expression. This is undesirable, as this variance most probably represents technical and not biological variation. Therefore, we deemed it necessary to also standardize the variance of each gene within each batch, which meant that we had to remove all studies that had only one sample, leaving us with 2,959 samples.

# 3 Competing Methods

## 3.1 Mutual Rank (*MR*)

An alternative way to measure co-expression is using the Mutual Rank (*MR*) which has been successfully used in the ATTED-II co-expression database [1]. For every gene i, the co-expression values to all other genes are ranked in descending order. We use $rank_i(j)$ to denote the rank of gene $j$ in terms of similarity to $i$. Note that $rank_i(i) = 0$. The *MR* value between two genes is calculated as the geometric mean of $rank_i(j)$ and $rank_j(i)$:

$$MR(\mathbf{x}_i, \mathbf{x}_j) = \sqrt{rank_i(j) \times rank_j(i)} \tag{1}$$

*MR* is expected to be more robust to spurious correlations caused by outliers (due to the geometric mean) and can also handle cases where a gene tends to have very high or very low correlations with many other genes, which bias the co-expression ranks leading to poorer performance for the $k$-NN classifier. We downloaded the pairwise *MR* values constructed from public RNA-Seq data for all *A. thaliana* genes from the ATTED-II website (`http://atted.jp/top_download.shtml`).

## 3.2 *GAAWGEFA*

For *GAAWGEFA* [2] we used the MATLAB code provided by the authors at `http://sampa.droppages.com/GAAWGEFA.html`. Because of the long runtime we did not tune any of the parameters of the algorithm and used the default settings as mentioned in the paper:

- Initial population size: 20
- Crossover Probability: 0.85
- Mutation Probability: 0.1
- Maximum iterations: 1000

After the 150-th iteration we enabled the extra option of early termination, i.e. we stopped the optimization if the fitness function had not increased by at least 1% between two consecutive generations. We did tune the number of nearest neighbors used in the $k$-NN classifier as previously.

# 4 Evaluation Modes

## 4.1 Cross-Validation Experiment

We used the $k$-Nearest Neighbors ($k$-NN) classifier to compare the function prediction performance of the different studied co-expression measures on all *A. thaliana* genes with at least one BP annotation. To counter the imbalance in the dataset, we restricted ourselves to GO terms that annotate at least 1% of the genes. Also, for the weight optimization stage of *MLC*, we randomly sampled an equal number of genes with and without each term. The optimal number of nearest neighbors ($k$) is a parameter of all methods. *MLC* has an extra regularization parameter $\alpha$ (equation 6, main document). We tuned the parameters of *MLC* independently for each GO term, while we selected the value of $k$ that maximized the mean performance over all tested GO terms for the other methods. Parameter tuning was done in a double 3-fold cross-validation loop [3] (Figure S2) using the term-centric *ROCAUC* as performance criterion. The inner loop was used to select the optimal parameter values and the outer loop to evaluate the performance of the tuned models on previously unseen genes.

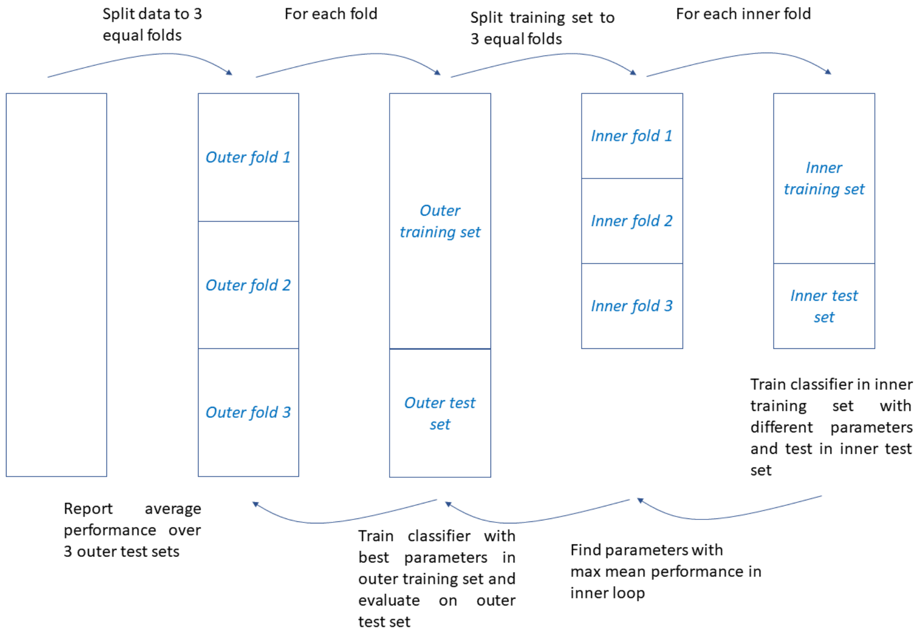


Figure S2: Schematic representation of a nested cross-validation loop.

## 4.2 CAFA3 Experiment

We also evaluated the same methods on the preliminary test set from CAFA3, released by the organizers in June 2017. This dataset contains 6,077 training and 137 test genes from *A. thaliana*. After ID mapping, we restricted ourselves only to the genes for which we had both expression data from ArrayExpress and *MR* values from the ATTED database and passed the filtering step described in section 2.1 (main document). This left us with 4,889 training genes and 90 test genes, annotated with 707 GO terms. We used the training set to tune the parameters of the tested methods using a 3-fold cross-validation loop. We removed the 10 rarest terms, as there were not enough training and testing genes in all folds. Then, we re-trained each method on the whole training set, using the optimal parameter values found with cross-validation and made predictions for the 697 remaining terms on the 90 test genes (reported as CAFA3 results). To assess the variability of the results, we performed 1,000 bootstraps, choosing at random with replacement 90 genes at each iteration and re-evaluating the mean term-centric performance. We used these bootstraps to construct 95% confidence intervals.

### 4.3 CAFA-$\pi$

Finally, we compared *MLC* to *MR* and *PCC* using data from the CAFA-$\pi$ challenge [4]. The goal of CAFA-$\pi$ was to make term-specific predictions for biofilm formation and motility in *Pseudomonas aeruginosa* and *Candida albicans*, which were then tested using genome-wide assays. For pseudomonas, the organizers of the challenge used two baseline methods that made use of an existing microarray dataset with 1,051 samples and *PCC* [5]. These baseline methods turned out to be the top-performing methods in the challenge. We compared our method and *MR* to these *PCC*-based baselines. *GAAWGEFA*, uses all available GO terms and is trained in a protein-centric way, so we did not test *GAAWGEFA* in this strictly term-centric setting.

As starting point, all methods use the genes that were already experimentally annotated with the function of interest (biofilm or motility) before the challenge (training genes). Note that these training genes are only of the positive class, however some of them were not validated as positives by the assay. Nevertheless, we still used all these genes as positive genes, to ascertain a fair comparison with methods implemented before the challenge. For the baseline methods, the posterior probability of a test gene being involved in the function was calculated as either the maximum *PCC* value between that gene and all training genes, or the average value of the top-10 most co-expressed training genes [4]. We applied the same strategy for *MR* and *MLC*. *MLC* requires a negative set during training, i.e. a set of genes known not be involved in the function of interest. In this case, we chose the negative set as a random subset of the genes for which expression data were available, but they were not screened for the function, so their involvement in the function is unknown both before and after the CAFA-$\pi$ challenge. For example, these could be essential genes whose knock-out is lethal. *MLC* has a parameter $\alpha$ which we previously tuned using cross-validation. This is not possible in this setting due to the small number of training genes, so we further tested MLC with two values of $\alpha$: one that performs sample selection (0.75, selecting fewer than 10% of the samples) and one that does not (0.25, selecting all samples).

# 5 Evaluation Measures

## 5.1 Unweighted and Weighted term-centric ROC AUC

In the setting used in this work, we are interested in finding the genes that perform a given function, so term-centric evaluation is the most interesting. As in the CAFA papers, we use the term-centric area under the ROC curve ($ROCAUC$). For every GO term $l$, we compute a $ROCAUC$ score for the binary problem of finding all test genes that are annotated with that term and then we report the average of that value over all GO terms. We also use the weighted version of this measure ($ROCAUC_w$), in which we calculate a weighted average, weighing each term by its Resnik Information Content ($IC(l)$) [6].

## 5.2 Protein-Centric Measures

For protein-centric evaluation, we use the F1 score ($F_{max}$) and the Semantic Distance ($S_{min}$) [7]. Similar to the CAFA evaluation, we start with the posterior probabilities for each pair of test gene and GO term. As these measures require hard predictions (i.e. either 0 or 1, not a posterior probability) we compute them for 21 equally-spaced thresholds from 0 to 1 (i.e. in steps of 0.05). We then only report the metric value at the optimal threshold for each measure (the maximum for the F1 and the minimum for the Semantic Distance) and for each evaluated algorithm [8].

## 6 Protein-Centric Results

We compared 5 methods: *PCC*, *PCC+MR*, *GAAWGEFA*, *MLC* and $MLC_G$ using 2 term-centric and 2 protein-centric evaluation measures. Term-centric performance is shown in Table 1 in the main document. Tables S1 and S2 show the protein-centric $F_{max}$ and $S_{min}$ for these methods on the cross-validation and CAFA3 datasets respectively. We observe that, according to both metrics, all methods achieve more or less equivalent protein-centric performance, with the exception of the *PCC*, which performs significantly worse (Supplementary Material 7). The *MR* seems to be consistently the top method, but the differences are very small and not statistically significant (See also Supplementary Material 7).

Table S1: Comparison of the protein-centric $F_{max}$ and $S_{min}$ of the tested methods using 3-fold cross-validation. For each method and metric the mean and standard error are shown. Higher $F_{max}$ and **lower** $S_{min}$ denote better performance.

| Method | $F_{max}$ | $S_{min}$ |
|---|---|---|
| *PCC* | $0.34 \pm 0.001$ | $19.12 \pm 0.11$ |
| *PCC + MR* | $0.36 \pm 0.001$ | $18.85 \pm 0.11$ |
| *GAAWGEFA* | $0.35 \pm 0.002$ | $18.97 \pm 0.10$ |
| *MLC* ($S_w$) | $0.35 \pm 0.001$ | $18.89 \pm 0.13$ |
| $MLC_G$ ($S_w$) | $0.35 \pm 0.003$ | $19.00 \pm 0.17$ |

Table S2: Comparison of the protein-centric $F_{max}$ and $S_{min}$ of the tested methods using the CAFA3 data. For each method and metric the mean and 95% confidence interval is shown. Higher $F_{max}$ and **lower** $S_{min}$ denote better performance.

| Method | $F_{max}$ | $S_{min}$ |
|---|---|---|
| *PCC* | 0.25 [0.22, 0.29] | 21.27 [18.78, 29.17] |
| *PCC + MR* | 0.27 [0.24, 0.30] | 21.18 [18.78, 28.99] |
| *GAAWGEFA* | 0.25 [0.22, 0.29] | 21.32 [18.90, 29.10] |
| *MLC* ($S_w$) | 0.26 [0.23, 0.29] | 21.56 [18.87, 29.11] |
| $MLC_G$ ($S_w$) | 0.27 [0.24, 0.31] | 21.27 [18.81, 29.22] |

# 7 Statistical Significance of Differences in Cross-Validation Performance

We use four evaluation metrics ($ROCAUC$, $ROCAUC_w$, $F_{max}$, $S_{min}$) to compare 5 methods ($PCC$, $PCC + MR$, $GAAWGEFA$, $MLC$ and $MLC_G$). We used the paired-sample $t$-test to compare all 10 different pairs of methods across the three cross-validation folds. This test tests the null hypothesis that the mean difference in performance between two methods across the three folds is not different from zero. For every combination of two methods and a metric we obtained a p-value, so in total we obtained 40 p-values. We corrected all these p-values jointly for multiple testing using the Benjamini-Hochberg method for controlling the False Discovery Rate (FDR). The results are listed in tables S3 to S6. *MR* and *MLC* are significantly better than *PCC* according to all four metrics.

Table S3: FDR-corrected p-values for the null hypothesis that the cross-validation $ROCAUC$ of two methods is not different from zero. Entries are colored in red if the row method is significantly worse than the column method (FDR $<$ 0.05) and in green if the row method is significantly better than the column method (FDR $<$ 0.05).

| | $PCC + MR$ | $GAAWGEFA$ | $MLC$ $(S_w)$ | $MLC_G$ $(S_w)$ |
|---|---|---|---|---|
| $PCC$ | 0.008 | 0.038 | 0.021 | 0.041 |
| $PCC + MR$ | | 0.088 | 0.272 | 0.180 |
| $GAAWGEFA$ | | | 0.066 | 0.175 |
| $MLC$ $(S_w)$ | | | | 0.372 |

Table S4: FDR-corrected p-values for the null hypothesis that the cross-validation $ROCAUC_w$ of two methods is not different from zero. Entries are colored in red if the row method is significantly worse than the column method (FDR $<$ 0.05) and in green if the row method is significantly better than the column method (FDR $<$ 0.05).

| | $PCC + MR$ | $GAAWGEFA$ | $MLC$ $(S_w)$ | $MLC_G$ $(S_w)$ |
|---|---|---|---|---|
| $PCC$ | 0.008 | 0.038 | 0.016 | 0.041 |
| $PCC + MR$ | | 0.088 | 0.229 | 0.189 |
| $GAAWGEFA$ | | | 0.041 | 0.178 |
| $MLC$ $(S_w)$ | | | | 0.155 |

Table S5: FDR-corrected p-values for the null hypothesis that the cross-validation $F_{max}$ of two methods is not different from zero. Entries are colored in red if the row method is significantly worse than the column method (FDR $<$ 0.05) and in green if the row method is significantly better than the column method (FDR $<$ 0.05).

| | $PCC + MR$ | $GAAWGEFA$ | $MLC$ $(S_w)$ | $MLC_G$ $(S_w)$ |
|---|---|---|---|---|
| $PCC$ | 0.038 | 0.113 | 0.038 | 0.155 |
| $PCC + MR$ | | 0.154 | 0.302 | 0.047 |
| $GAAWGEFA$ | | | 0.258 | 0.180 |
| $MLC$ $(S_w)$ | | | | 0.180 |

Table S6: FDR-corrected p-values for the null hypothesis that the cross-validation $S_{min}$ of two methods is not different from zero. Entries are colored in red if the row method is significantly worse than the column method (FDR $<$ 0.05) and in green if the row method is significantly better than the column method (FDR $<$ 0.05).

| | $PCC + MR$ | $GAAWGEFA$ | $MLC$ $(S_w)$ | $MLC_G$ $(S_w)$ |
|---|---|---|---|---|
| $PCC$ | 0.021 | 0.066 | 0.038 | 0.155 |
| $PCC + MR$ | | 0.119 | 0.223 | 0.138 |
| $GAAWGEFA$ | | | 0.246 | 0.181 |
| $MLC$ $(S_w)$ | | | | 0.229 |

## 8 *MLC* does not pick up artificial information

We started from the label matrix $Y \in \{0,1\}^{N \times L}$, where $L$ is the total number of GO terms. Column $l$ of $Y$ represents to the vector $\mathbf{y}(l)$ that contains a 1 at the rows that correspond to the genes annotated with $l$. We randomly permuted the rows of the label matrix, so that each gene is assigned to a random set of GO terms, but both the consistency of the ontology graph and the GO term frequencies are preserved. Then we ran both *PCC* and term-specific *MLC* on this random dataset, including the tuning of the parameters using a double-loop cross-validation as described in Supplementary Material 4. We found that both *PCC* and *MLC* achieved a mean term-centric *ROCAUC* of 0.5 (i.e. equal to random guessing). This shows that in absence of real structure in the data, sample selection or re-weighing does not artificially generate information (i.e. false positives are controlled).

## 9 False positive rates for general and specific terms

To explain why *MLC* performs better for specific terms than for general ones, we compared the ROC curves of the 20% most specific terms to those of the 20% most general ones. Term specificity was measured by the Resnik Information Content [6]. To get an indication of the general behaviour pattern of *MLC*, we averaged all ROC curves in each of the two groups of GO terms. Figure S3 shows these two average curves. We observed that near the point $(0, 0)$, which corresponds to the genes that *MLC* classified as positive with high posterior probability, the average ROC curve of the specific terms is increasing a lot more sharply than the one of the general terms which is smoother. This means that genes for which *MLC* is most confident that are positive are indeed enriched with positive labels for the specific terms. The curve of the general terms is increasing more smoothly, meaning that for those terms, *MLC* is scoring a lot of negative genes highly, resulting in more false positive predictions.

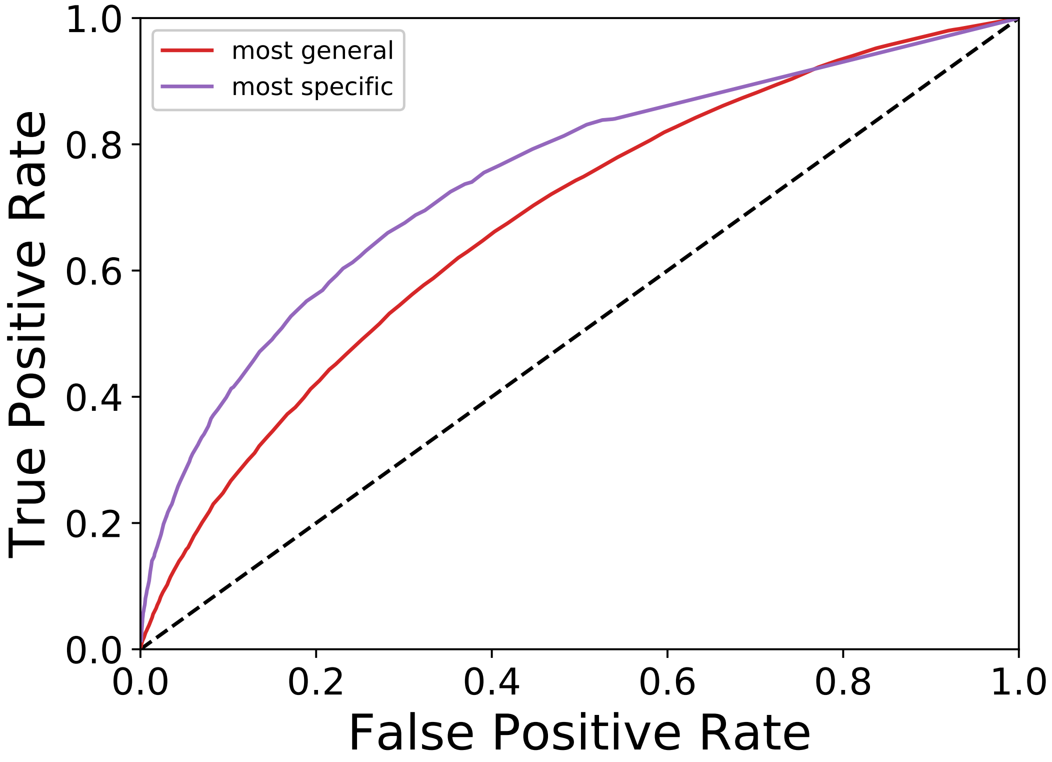


Figure S3: Term-centric ROC curves averaged over the terms with the 20% highest IC (purple) and with the 20% lowest IC (red). The $y = x$ line which represents the expected ROC of a random classifier is shown with a black dashed line.

# 10 Performance as a function of term specificity

For each pair of methods, we calculate the percent difference in $ROCAUC$ between them for each GO term. Tables S7 and S8 show the Spearman correlation of these values with the Information Content and the maximum path length to the ontology root of each term respectively. Moreover, in Figures S4 and S5 we plot these differences for each pair. From these we can clearly conclude that term-specific $MLC$ is the best of all tested methods at predicting specific terms.

Table S7: Spearman correlation of the % difference in performance between the method in each row and the one each column with term Resnik Information Content. Statistically significant values (FDR < 0.05) are shown in bold.

| | $PCC$ | $PCC + MR$ | $GAAWGEFA$ | $MLC$ | $MLC_G$ |
|---|---|---|---|---|---|
| $PCC$ | - | -0.028 | -0.041 | **-0.174** | -0.055 |
| $PCC + MR$ | 0.028 | - | 0.016 | **-0.165** | 0.029 |
| $GAAWGEFA$ | 0.041 | -0.016 | - | **-0.163** | 0.003 |
| $MLC$ | **0.174** | **0.165** | **0.163** | - | **0.164** |
| $MLC_G$ | 0.055 | -0.029 | -0.003 | **-0.164** | - |

Table S8: Spearman correlation of the % difference in performance between the method in each row and the one each column with term path length to the ontology root. Statistically significant values (FDR < 0.05) are shown in bold.

| | $PCC$ | $PCC + MR$ | $GAAWGEFA$ | $MLC$ | $MLC_G$ |
|---|---|---|---|---|---|
| $PCC$ | - | -0.007 | -0.035 | **-0.259** | -0.035 |
| $PCC + MR$ | 0.007 | - | 0.022 | **-0.246** | 0.015 |
| $GAAWGEFA$ | 0.035 | -0.022 | - | **-0.244** | -0.018 |
| $MLC$ | **0.259** | **0.246** | **0.244** | - | **0.255** |
| $MLC_G$ | 0.035 | -0.015 | 0.018 | **-0.255** | - |

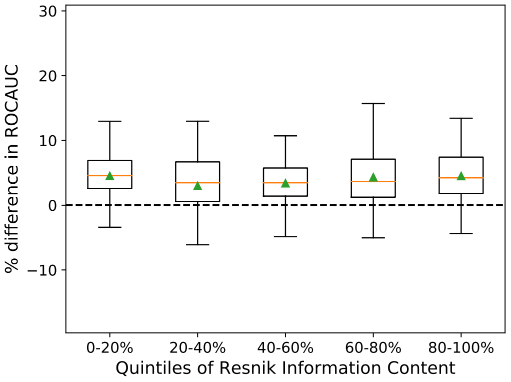


(a) $MR$ - $PCC$

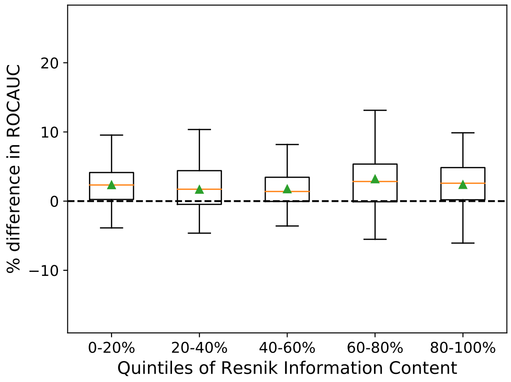


(b) $GAAWGEFA$ - $PCC$

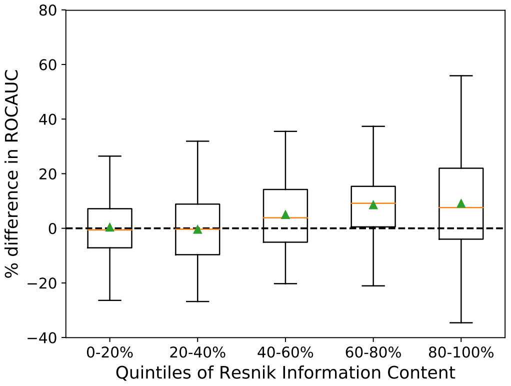


(c) $MLC$ - $PCC$

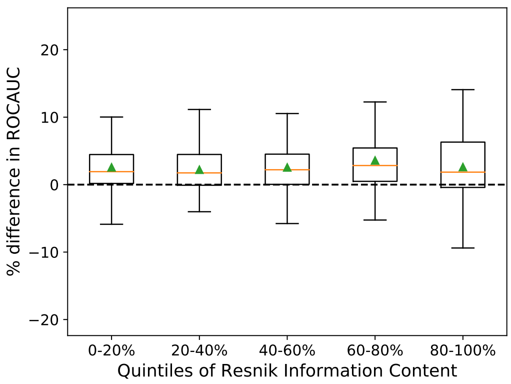


(d) $MLC_G$ - $PCC$

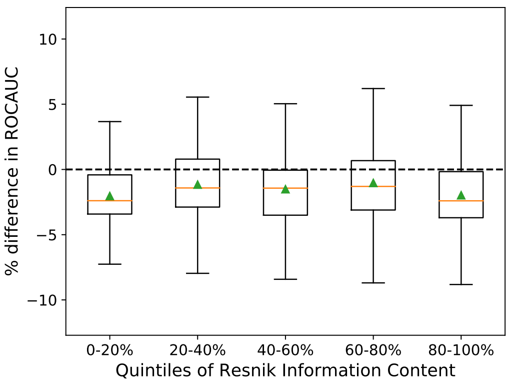


(e) $GAAWGEFA$ - $MR$

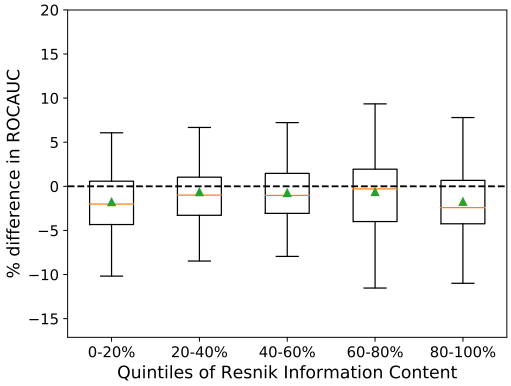


(f) $MLC_G$ - $MR$

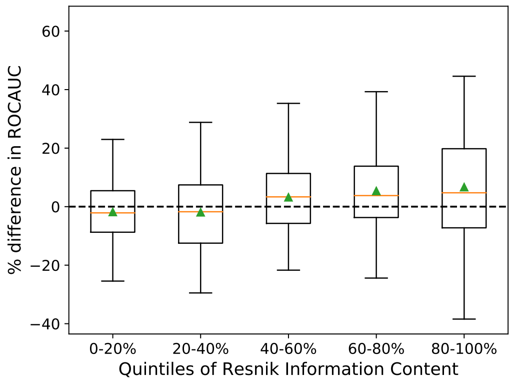


(g) *MLC - GAAWGEFA*

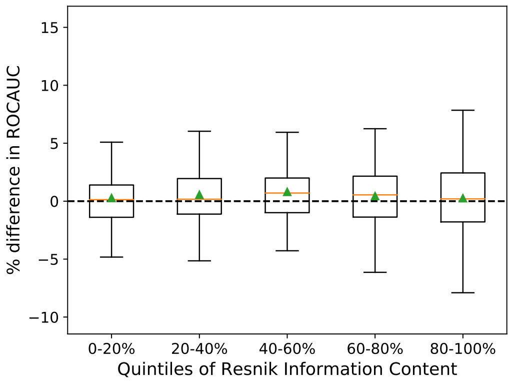


(h) $MLC_G$ - *GAAWGEFA*

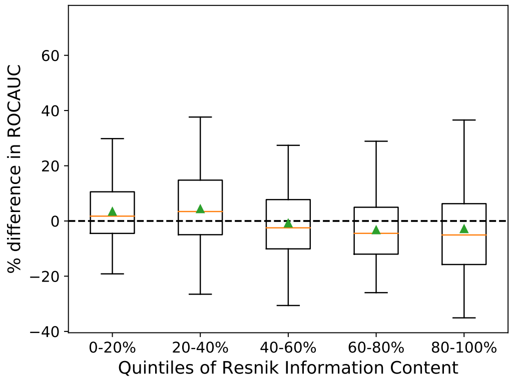


(i) $MLC_G$ - *MLC*

Figure S4: Percent difference in $ROCAUC$ of all pairs of methods as a function of Resnik Information Content. For each set of terms in each quintile of Information Content, the corresponding box includes the two middle quartiles of the percent difference for these terms. An orange line denotes the median and a green triangle the mean. The error bars extend to 1.5 times the range of the two middle quartiles.

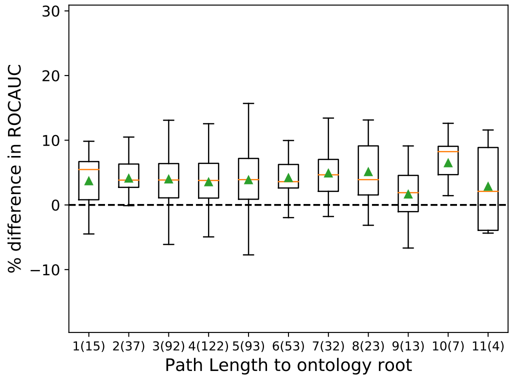


(a) *MR - PCC*

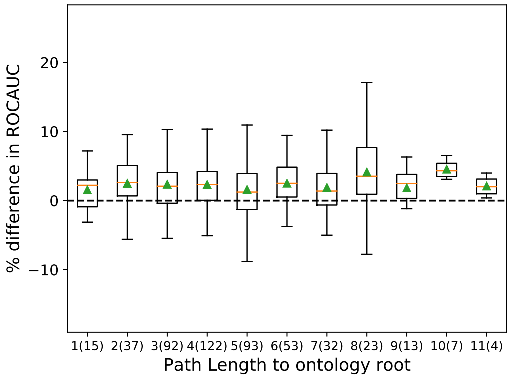


(b) *GAAWGEFA - PCC*

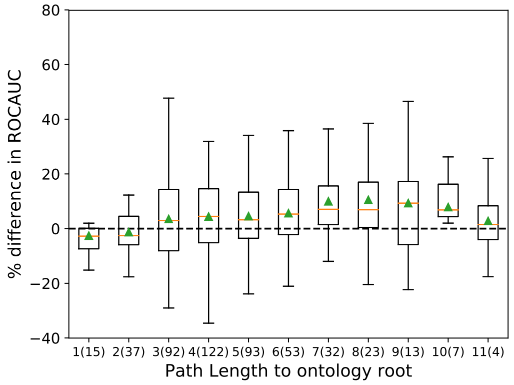


(c) *MLC - PCC*

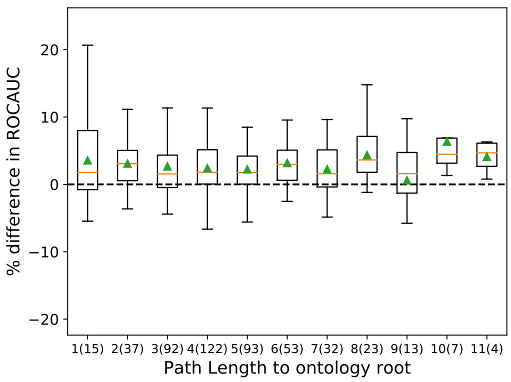


(d) $MLC_G$ - *PCC*

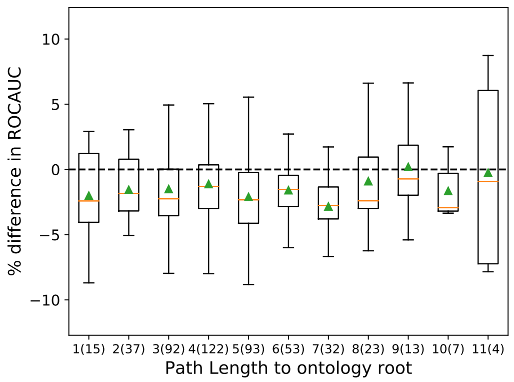


(e) *GAAWGEFA - MR*

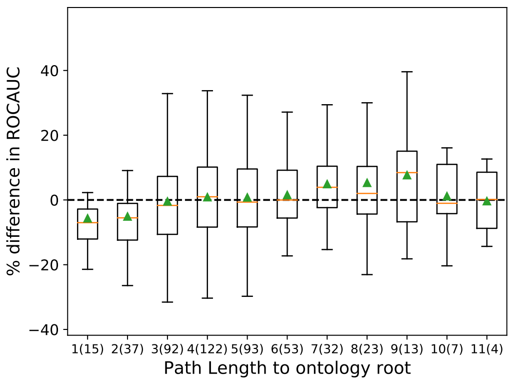


(f) *MLC - MR*

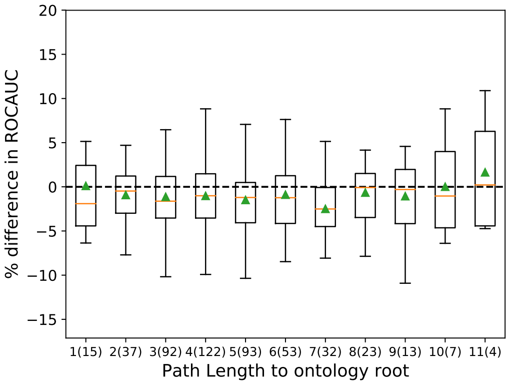


(g) $MLC_G$ - $MR$

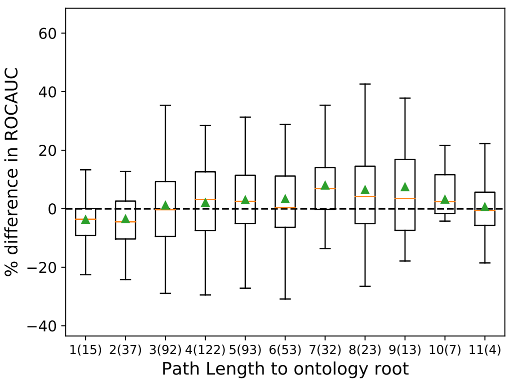


(h) $MLC$ - $GAAWGEFA$

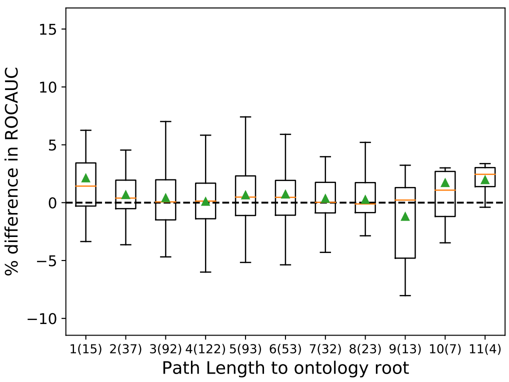


(i) $MLC_G$ - $GAAWGEFA$

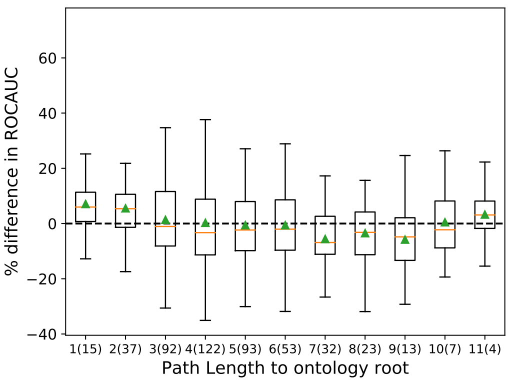


(j) $MLC_G$ - $MLC$

Figure S5: Percent difference in $ROCAUC$ of all pairs of methods as a function of path length to the ontology root. For each set of terms with a given path length, the corresponding box includes the two middle quartiles of the percent difference for these terms. An orange line denotes the median and a green triangle the mean. The error bars extend to 1.5 times the range of the two middle quartiles. The numbers in parentheses next to each path length level denote the number of terms in the dataset at that level.

## 11 Only reducing the number of samples does not boost $PCC$ performance

We tested the performance of the $PCC$ on a randomly selected subset of 250 samples out of the original 2,959 samples. We repeated this experiment with 5 different subsets of the same size and in all cases we found the mean $ROCAUC$ to be similar to that of $PCC$ with all samples ($0.69 \pm 0.01$). This shows that simply reducing the number of samples is not enough to get a performance boost and that we really need to identify the relevant samples for each GO term.

# 12 Comparison of *GAAWGEFA* and *MLC* weights

In Figure S6a we show the distribution of the weight values of the "average" weight profile of *MLC*. To obtain that, we sort all term-specific weight profiles from the smallest to the largest value and then calculate the average weight value at each rank over all term profiles. Figure S6b shows the weight distribution of the *GAAWGEFA*, which is not term-specific. We calculated the *PCC* between each term-specific profile and the *GAAWGEFA* profile and also between each term-specific profile and the $MLC_G$ profile. Figure S7 shows the distribution of these two similarities. In conclusion, the weights learned by *GAAWGEFA* were not correlated to the ones learned by *MLC* or $MLC_G$.

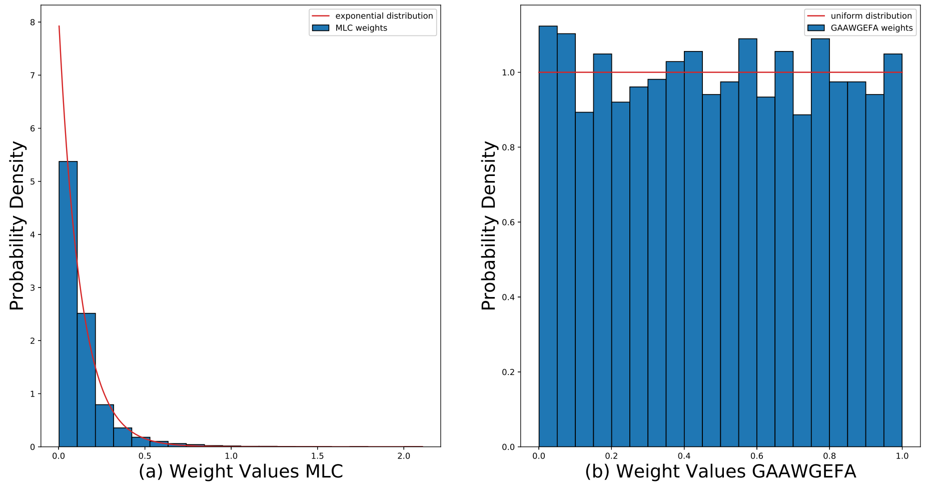


Figure S6: Histogram ($y$-axis) of the sample weight values ($x$-axis) for the average *MLC* profile (a) and the GAAWGEFA profile (b). The best fitting exponential distribution (a) and uniform distribution (b) are shown in red.

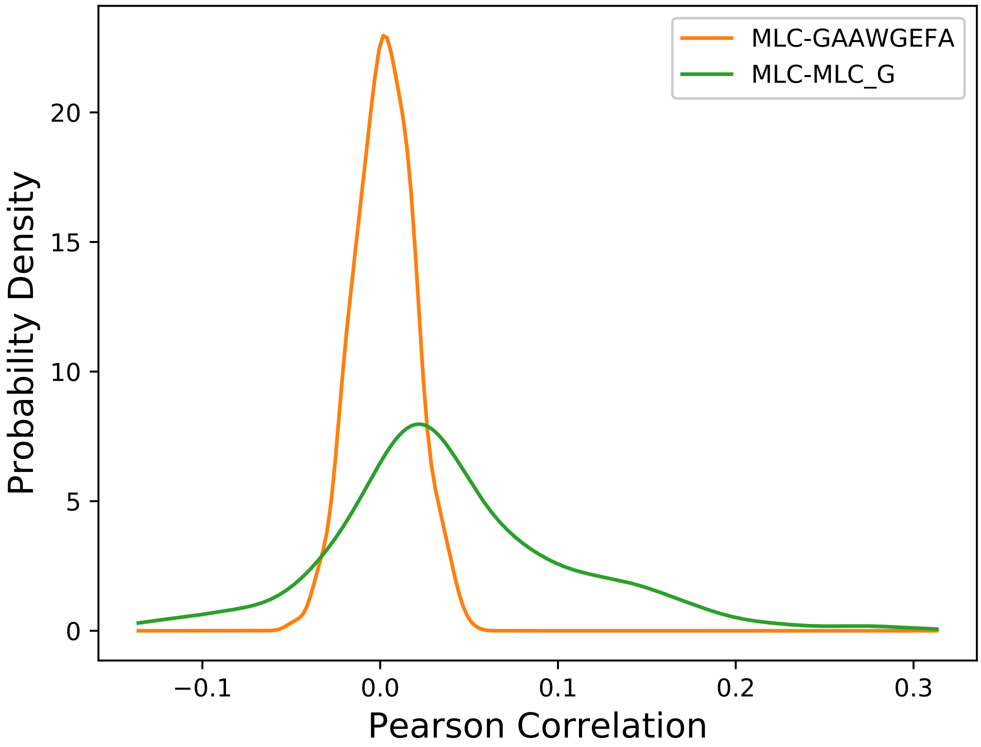


Figure S7: Distribution of the pairwise Pearson correlation values between the $MLC$-derived GO-term-specific weight profiles and the weight profile of $MLC_G$ (green) and $GAAWGEFA$ (orange). The $x$ axis corresponds to the correlation values and the $y$ axis to the probability density.

## 13 Samples from the same study tend to get either selected or not selected together

We tested whether samples from a relevant batch are more often selected together than expected. The null hypothesis in this case is that which batch a sample comes from is independent of whether it gets selected for a particular term. Under the null hypothesis, the number of samples that get selected from each batch are proportional to the total number of samples in the batch. This can be modelled using a multinomial distribution: Each batch is viewed as a categorical variable and under the null hypothesis the probability of observing a sample from that batch is equal to the number of samples in that batch divided by the total number of samples. In each cross-validation fold, we looked at the GO terms for which *MLC* selected fewer than 1,000 samples. For each of those terms, we performed the multinomial test, testing whether more or fewer samples were selected from the same batches than expected under the null hypothesis. To save computation time, we did not perform an exact test, but rather two approximations, one using the chi-squared test and one using Monte Carlo sampling (drawing 1 million samples per test), both implemented in the R package XNomial (`https://cran.r-project.org/package=XNomial`). With both approximations, we found that for at least 97% of the terms, the observed batch frequencies were significantly different from what would be expected by the global batch frequencies with a False Discovery Rate of 0.05 (Table S9). This shows that batches indeed tend to be either enriched or depleted with selected samples for each GO term.

Table S9: Results of two approximations of the multinomial test with null hypothesis that the sample selection is independent of which batches samples come from. For each cross-validation fold, we show the number and fraction (in parentheses) of the terms for which the null hypothesis was rejected with a False Discovery Rate of 0.05.

| **Fold** | **Terms tested** | **Terms significant by Monte Carlo** | **Terms significant by $\chi^2$** |
|---|---|---|---|
| 0 | 176 | 175 (99.4%) | 171 (97.2%) |
| 1 | 187 | 186 (99.5%) | 183 (97.9%) |
| 2 | 164 | 163 (99.4%) | 163 (99.4%) |

## 14 Evaluation on simulated data

We simulated an expression dataset with 7,000 genes and 3,000 samples, which is similar to the size of our real *A. thaliana* dataset. 15% of these genes were annotated with a GO term of interest (target class). The remaining 85% were evenly split between two other GO terms (background classes 1 and 2). The genes of each of the three classes had a distinct expression pattern: For the target class, the genes within this class have an expression pattern that changes as a noisy sine wave for the first $x$ samples. The expression values for the remaining $n - x$ samples are drawn from a Gaussian noise distribution. The background classes had similar expression patterns, but the starting location, frequency and phase of the sine were different for both classes. Note that the number of informative samples, $x$, is the same for all three classes, but which samples are informative is different for each of them. To model different basal expressions, each gene was given a random mean expression drawn from a Gaussian distribution (N(0, 3)). Examples of the three classes are shown in Figure S8.

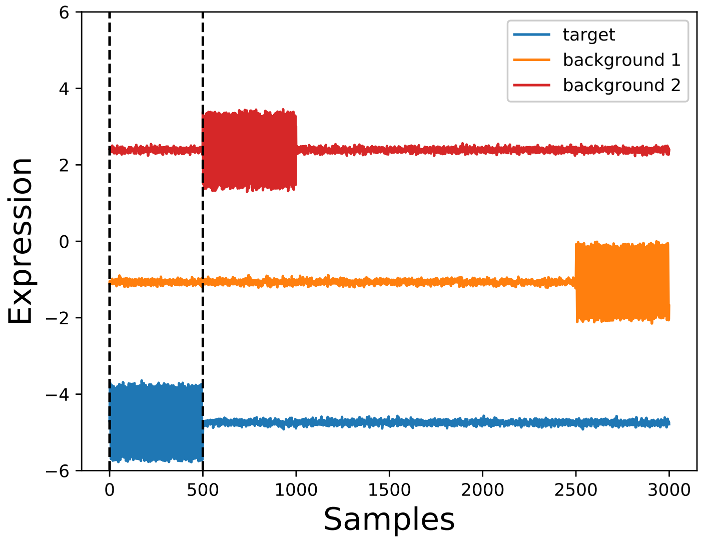


Figure S8: Examples of the generated expression profiles from the three classes, where the relevant number of samples $x$ is equal to 500. The gene expression is on the $y$ axis and the samples on the $x$ axis. The example from the target class is shown in blue and the ones from the other classes in orange and red. Two black dashed lines denote the relevant samples for the target class.

We split the dataset into a training (5,000 genes), validation (1,000 genes) and test set (1,000 genes) in a stratified way. We used the validation set to tune the $\alpha$ parameter of *MLC* and then trained on both the training and validation set and tested on the test set. For the *PCC* (which uses all samples) and the ground-truth *PCC* (which uses only samples 1 to $x$), we also used the training and validation set as training genes. The number of nearest neighbors ($k$) was set to 30 for all three methods.

We then varied the number of relevant samples ($x$) from 10 to 500 and repeated the experiment 5 times for each value of $x$ instantiated, where each time we generate a different simulated dataset.

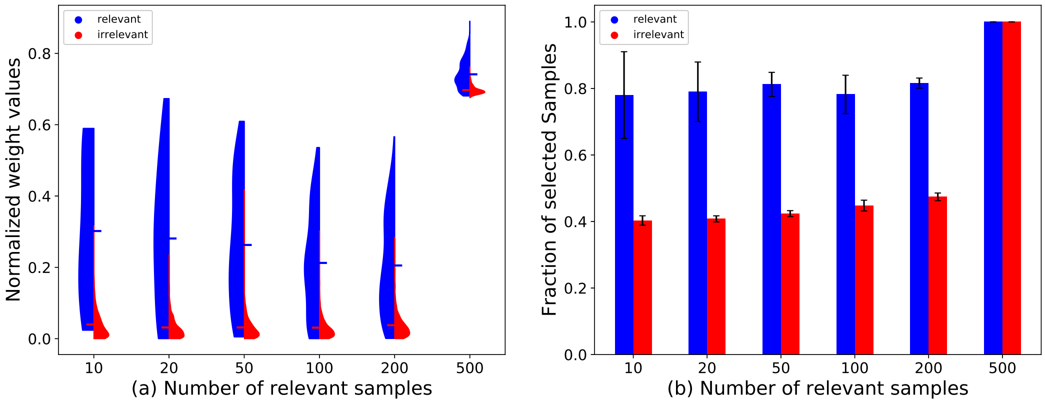


Figure S9: (a) Distribution of weights learned by *MLC* ($y$ axis, scaled in the range [0-1]) for the relevant samples (blue) and the rest of the samples (red) for different numbers of relevant samples ($x$ axis) on simulated data. The mean of each distribution is denoted by a horizontal line. (b) Fraction of the samples that were selected by *MLC* ($y$ axis) for the relevant samples (blue) and the remaining samples (red) for different numbers of relevant samples ($x$ axis). The error bars denote the standard deviation over 5 repetitions.

## 15 Weight profile

Figure S10 shows the weight profile that *MLC* learned for term GO:1903047 (mitotic cell cycle process).

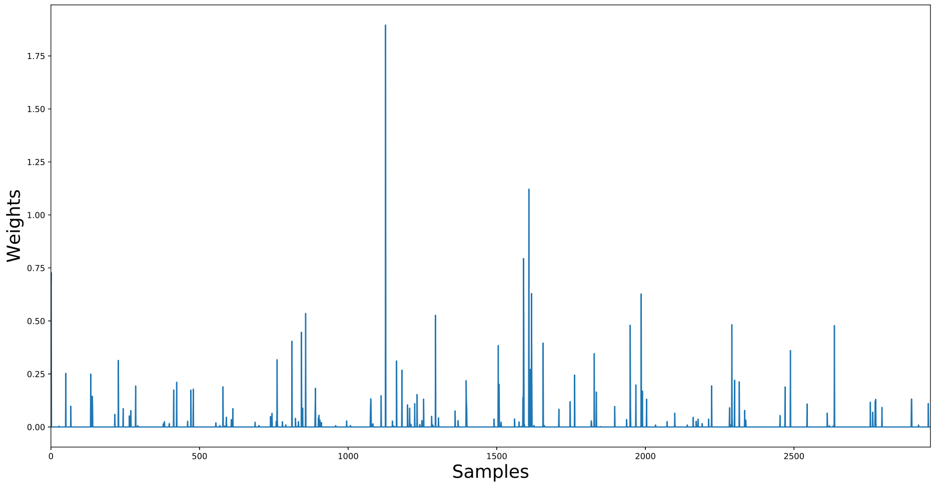


Figure S10: The weight profile learned by *MLC* for term GO:1903047. On the $x$ axis are the 2,959 RNA-Seq samples and on the $y$ axis the weight value of each sample.

# 16 The weights learned by $MLC$ are consistent with the ontology structure and the existing annotations

We compared the sample weights learned by $MLC$ between parent-child GO term pairs in the following three cases: 1) the parent has only one child term and both parent and child annotate exactly the same genes, 2) the parent has only one child term but annotates more genes than its child, and 3) the parent has exactly two children, meaning that the parent annotates the union of the genes of its children. In the first case, the weight profiles for parent and child are identical (mean Pearson correlation of 1). In the second case, the profiles are similar but not identical (mean Pearson correlation of profiles 0.47). The difference in mean similarity between the two groups is statistically significant (permutation p-value $< 10^{-5}$). Furthermore, the larger the difference in number of extra genes of the parent term, the smaller the profile correlation (Spearman $\rho = -0.53$, $CI_{95\%} = [-0.65, -0.40]$). The profile similarities are even smaller in the third case (mean of 0.36) and significantly smaller than those of case 2 (permutation p-value = 0.0002). This is expected as in this case the parent contains two distinct sets of genes that correspond to two different biological processes. Again, we found a negative correlation between the number of different genes and the profile similarity of pairs (Spearman $\rho = -0.47$, $CI_{95\%} = [-0.61, -0.31]$).

To generalize this finding, we hierarchically clustered the GO terms (complete linkage, Jaccard distance between the gene sets associated with each GO term cutoff of 0.6). The resulting clusters are shown in Figure S11 along with the pairwise distances of the GO terms. 64 out of 176 clusters contained at least three terms. For each of these 64 clusters, we randomly sampled 10,000 equal-sized sets of GO terms and calculated the mean pairwise similarity in those sets to calculate a permutation p-value. For 62 out of these 64 clusters we found that the pairwise weight-profile similarities of their members (Figure S2, SM2) are significantly higher than random with a False Discovery Rate of 0.05. Also, pairwise profile similarities are positively correlated with the pairwise Resnik semantic similarity of GO terms (Spearman $\rho = 0.16$, $CI_{95\%} = [0.15, 0.17]$). Based on these observations, we conclude that sample weights reflect the gene annotations of each term.

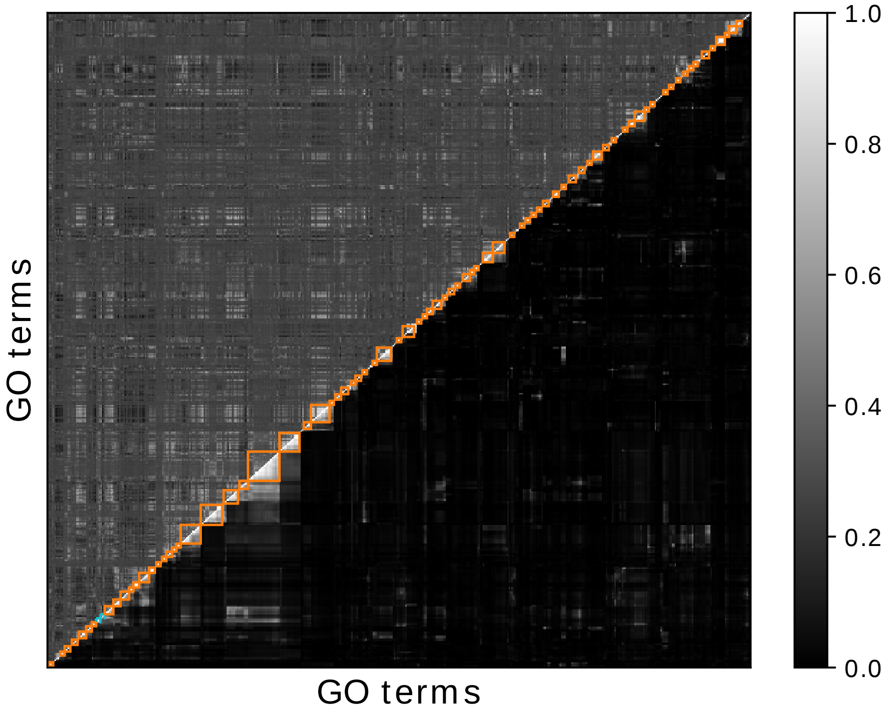


Figure S11: Pairwise Jaccard similarities (below the anti-diagonal) and weight profile similarities (Pearson correlations, above the anti-diagonal) of the tested GO terms. Both the $x$ and $y$ axes show the GO terms ordered so that terms in the same cluster are adjacent. The grey-scale indicates the similarity, with dark being low and bright being high similarity. The profile correlations have been scaled so that they are in the range [0, 1]. The squares highlight the clusters containing at least 3 terms, (cutting the dendrogram at a Jaccard distance threshold of 0.6 when using complete linkage). Light blue boxes indicate the clusters that are not significantly enriched with terms with similar weights and orange-colored clusters are significantly enriched after FDR correction.

# 17 CAFA-$\pi$ Results

*PCC*, *MR* and *MLC* achieve similar *ROCAUC* (around 0.6 for both biofilm and motility, Table S10)

Table S10: *ROCAUC* achieved by *PCC*, *MR* and *MLC* at the CAFA-$\pi$ dataset for biofilm formation and motility in *Pseudomonas aeruginosa*. The highest performance per column is shown in bold.

| **Method** | **ROC AUC Biofilm** | **ROC AUC Motility** |
|---|---|---|
| PCC - top 1 | 0.56 | 0.56 |
| PCC - top 10 | 0.58 | **0.60** |
| MR - top 1 | 0.57 | 0.58 |
| MR - top 10 | **0.60** | **0.60** |
| MLC - top 1 ($\alpha = 0.25$) | 0.56 | 0.56 |
| MLC - top 10 ($\alpha = 0.25$) | 0.58 | **0.60** |
| MLC - top 1 ($\alpha = 0.75$) | 0.57 | 0.54 |
| MLC - top 10 ($\alpha = 0.75$) | 0.59 | 0.56 |

Again, the samples selected by *MLC* were informative: For motility, the most informative sample was a phhR mutant. phhR is a transcription factor which – under certain conditions – regulates the biosynthesis of a molecule called PQS (pseudomonas quinolone signal) [4] which has been shown to repress motility [5]. Among a few wild-type samples, in the top-10 highest scored we found three samples from the same study that studied the effect of Phosphorus on motility [6]. For biofilm formation, 4 of the 10 highest-scored samples were isolates from cystic fibrosis patients, where Pseudomonas is known to produce biofilms [7]. Also samples from a BF8 treatment (inhibits biofilm formation, [8]) and a hydrogen peroxide treatment (promotes biofilm formation in other bacteria,[9]) were highly scored, as well as three sulfur starvation samples from the same study.

Moreover, we had a look at genes that were highly scored by *MLC* for biofilm but were not found by the screen to be involved in it (i.e. false positives of our method). Among the 8 top-scored false positives were 4 genes that are consecutive in the genome (so very likely to be part of the same operon), namely PA0088, PA0089, PA0090 and PA0091. Two out of these genes (PA0089 and PA0090) are annotated with the KEGG pathway for biofilm formation (pae02025). This makes it probable that the other two genes are also members of the same pathway. For instance, it is possible that these genes participate in biofilm formation under different conditions from those used at the experimental screening during CAFA-$\pi$. For motility, the highest-scored false positive was PA2496, a gene that has been found to be down-regulated in knockouts of the gene HapZ, which mediates motility [10].

These show that the CAFA-$\pi$ is not a perfect ground truth, as it reflects only one experimental condition, whereas our method can find good candidate genes for a particular process under various conditions.

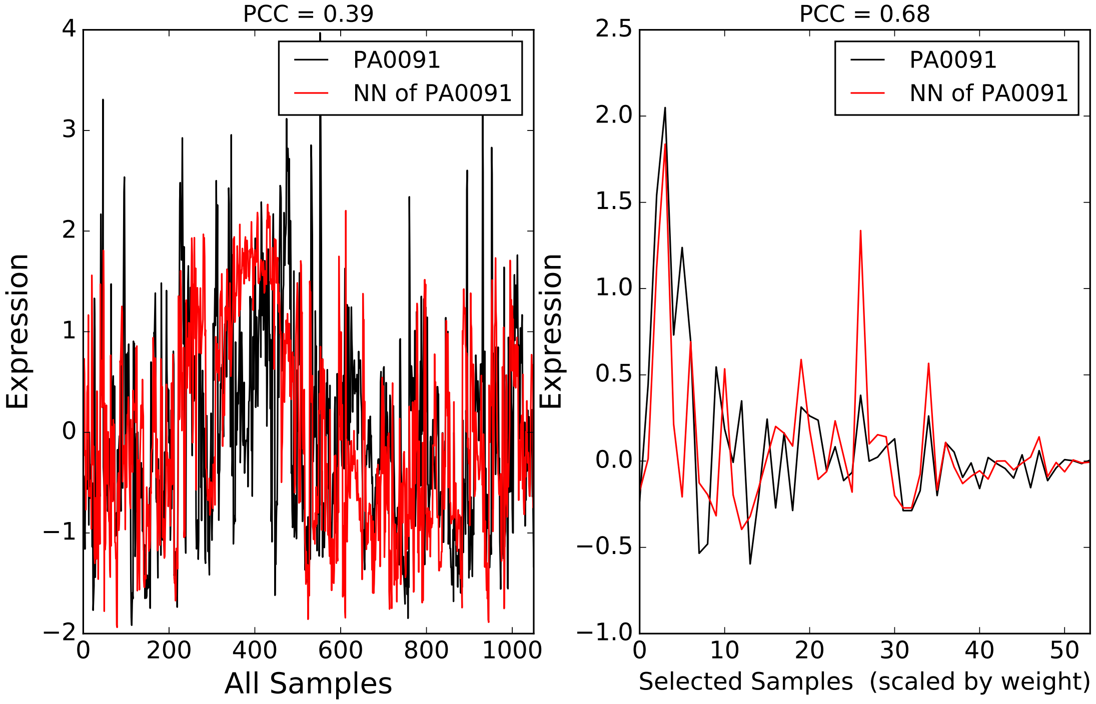


Figure S12: (Left) In black, the expression profile ($y$ axis) of gene PA0091 over the 1,051 samples of the dataset ($x$ axis). In red, the profile of the training gene with the highest $PCC$ to PA0091 over all the samples. The samples were ordered using hierarchical clustering on all genes, so that similar samples are next to each other. (Right) In black, the expression profile of PA0091 ($y$ axis) on the samples that were selected by $MLC$ ($x$ axis). In red, the profile of the training gene with the highest $PCC$ to PA0091 over only these samples. The samples are ordered in decreasing weight order and the expression is multiplied by that weight.

# References


[1] Yuichi Aoki et al. “ATTED-II in 2016: A plant coexpression database towards lineage-specific coexpression”. In: *Plant and Cell Physiology* 57.1 (2016), e5. ISSN: 14719053. DOI: 10.1093/pcp/pcv165.

[2] Shubhra Sankar Ray and Sampa Misra. “Genetic algorithm for assigning weights to gene expressions using functional annotations”. In: *Computers in Biology and Medicine* (2019). ISSN: 18790534. DOI: 10.1016/j.compbiomed.2018.11.011.

[3] Sudhir Varma and Richard Simon. “Bias in error estimation when using cross-validation for model selection”. In: *BMC Bioinformatics* 7.1 (2006), p. 91. ISSN: 14712105. DOI: 10.1186/1471-2105-7-91.

[4] Naihui Zhou et al. “The CAFA challenge reports improved protein function prediction and new functional annotations for hundreds of genes through experimental screens”. In: *bioRxiv* (2019). DOI: 10.1101/653105. eprint: https://www.biorxiv.org/content/early/2019/05/29/653105.full.pdf. URL: https://www.biorxiv.org/content/early/2019/05/29/653105.

[5] J. Tan et al. “Unsupervised Extraction of Stable Expression Signatures from Public Compendia with an Ensemble of Neural Networks”. In: *Cell Syst* 5.1 (July 2017), pp. 63–71.

[6] Philip Resnik. “Using Information Content to Evaluate Semantic Similarity in a Taxonomy”. In: *roceedings of the 14th international joint conference on Artificial intelligence - Volume 1 - IJCAI'95* 1 (1995), p. 6. ISSN: 1045-0823. DOI: 10.1.1.55.5277. arXiv: 9511007 [cmp-lg]. URL: http://arxiv.org/abs/cmp-lg/9511007.

[7] Wyatt T. Clark and Predrag Radivojac. “Information-theoretic evaluation of predicted ontological annotations”. In: *Bioinformatics* 29.13 (2013), pp. i53–i61. ISSN: 13674803. DOI: 10.1093/bioinformatics/btt228.

[8] Yuxiang Jiang et al. “An expanded evaluation of protein function prediction methods shows an improvement in accuracy”. In: *Genome biology* 17.1 (2016), p. 184. ISSN: 1474-760X. DOI: 10.6084/m9.figshare.2059944. arXiv: 1601.00891. URL: http://arxiv.org/abs/1601.00891.
